# Fatores de Risco, Manejo e Evolução após Primeiro Infarto Agudo do Miocárdio: Um Estudo de Mundo Real Comparando Coortes de Mulheres e Homens na Rede TriNetX

**DOI:** 10.36660/abc.20230692

**Published:** 2024-10-17

**Authors:** Camila Mota Guida, Eduardo Juvenal de Souza, Leandro Menezes Alves da Costa, Thiago Luis Scudeler, Rafael Amorim Belo Nunes, Gustavo Bernardes de Figueiredo Oliveira

**Affiliations:** 1 Instituto Dante Pazzanese de Cardiologia São Paulo SP Brasil Instituto Dante Pazzanese de Cardiologia, São Paulo, SP – Brasil; 2 Hospital Alemão Oswaldo Cruz São Paulo SP Brasil Hospital Alemão Oswaldo Cruz, São Paulo, SP – Brasil

**Keywords:** Infarto Agudo do Miocárdio, Fatores de Risco, Doença Cardiovascular na Mulher

## Abstract

**Fundamento:**

Estudos de coorte internacionais têm consistentemente demonstrado ao longo das últimas décadas um prognóstico desfavorável em pacientes do sexo feminino após o primeiro infarto agudo do miocárdio. No entanto, dados nacionais sobre esse tema são limitados.

**Objetivos:**

O presente estudo tem como objetivo comparar coortes nacionais de homens e mulheres hospitalizados devido ao primeiro infarto agudo do miocárdio (IAM), examinando os desfechos a longo prazo.

**Métodos:**

Estudo retrospectivo, observacional, com dados de mundo real extraídos da plataforma global TriNetX, incluindo pacientes de ambos os sexos com diagnóstico confirmado de IAM por classificação internacional de doenças (CID), versão 11, código I21. O nível de significância estatística adotado na análise foi de 5% (0,05). O desfecho primário avaliado foi composto por óbito, nova hospitalização por IAM, procedimentos de revascularização miocárdica, ou insuficiência cardíaca após fase hospitalar e com seguimento de 5 anos.

**Resultados:**

Foram avaliados dados de 29.041 pacientes, dos quais 11.284 (38,4%) eram mulheres. A idade média das populações feminina e masculina foi, respectivamente, 64,4 e 59,8 anos. O grupo de mulheres apresentou maior ocorrência do desfecho combinado de óbito, nova hospitalização por IAM, procedimentos de revascularização miocárdica, ou insuficiência cardíaca após fase hospitalar e com seguimento de 5 anos (OR 1.058; IC 1.005 - 1.113; p = 0,03).

**Conclusão:**

Nesta grande coorte brasileira, o sexo feminino foi associado a maior ocorrência de eventos cardiovasculares em período de 5 anos após a alta hospitalar.

## Graphical abstract

**Figure:**
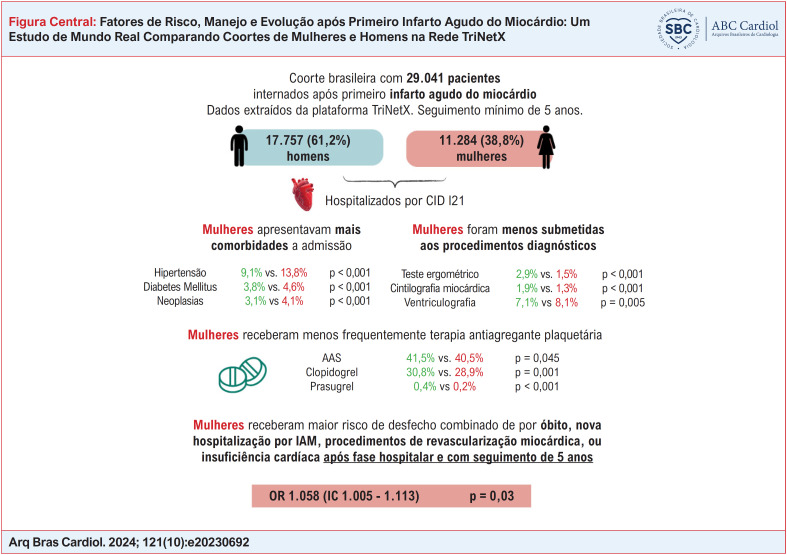
Estudo de mundo real comparando coortes de mulheres e homens na rede TriNetX.

## Introdução

Atualmente, a doença aterosclerótica coronariana é a principal causa de morte e perda de anos de vida ajustado por incapacidade ( *DALY - Disability Adjusted Life Years)* mundialmente, sendo responsável por quase 7 milhões de mortes e 129 milhões de *DALYs* por ano.^1^ No Brasil, especificamente, cerca de 13% dos óbitos podem ser atribuídos à doença arterial coronariana (DAC), conforme dados de 2017.^2^

A despeito das melhorias significativas na mortalidade cardiovascular em mulheres nas últimas duas décadas, a doença coronariana permanece pouco estudada, subdiagnosticada, e subtratada nesse grupo.^3 , 4^ A disparidade entre homens e mulheres está presente em todos os contextos: atendimento pré-hospitalar; tempo transcorrido até a procura por atendimento emergencial; manifestações clínicas do evento agudo; fatores de risco identificados; características das lesões coronarianas; tempo de início do tratamento específico; indicação de revascularização; cuidados pós-alta hospitalar, e no prognóstico tanto em curto quanto longo prazos.^5 - 10^ A baixa representatividade na base cientifica que norteia as condutas cardiológicas também se destaca como possível contribuinte para a discrepância observada.^11 - 16^

Desse modo, torna-se imprescindível a geração de conhecimento sobre os fatores de risco, os sinais e sintomas, as características angiográficas, o manejo hospitalar, e os fatores prognósticos entre mulheres e homens, em especial no contexto do primeiro IAM, cenário de significativa morbidade e mortalidade e que atinge pacientes muitas vezes vulneráveis pelo desconhecimento do próprio risco cardiovascular.

## Objetivos

Com base nas lacunas de evidência nacional e contemporânea em comparações robustas entre mulheres e homens com primeiro IAM, e pela relevância do aspecto de equidade em saúde na população brasileira, estabelecemos os seguintes objetivos: 1. Comparação entre mulheres e homens diagnosticados com o primeiro IAM quanto ao risco da ocorrência dos desfechos cardiovasculares compostos após a fase hospitalar até 5 anos (óbito, nova hospitalização por IAM, novos procedimentos de revascularização miocárdica, ou insuficiência cardíaca); 2. Comparação do perfil clínico contemporâneo quanto às comorbidades e apresentação clínica no contexto do primeiro IAM.

## Métodos

### Delineamento do estudo

Estudo do tipo observacional, de coorte retrospectiva e análise transversal, em mulheres e homens com diagnóstico confirmado de IAM. Utilizamos a base de dados da plataforma global colaborativa de evidência de mundo real TriNetX, uma rede de pesquisa em saúde global, que é alimentada por atualizações semanais dos dados registrados em sistemas de prontuários médicos eletrônicos anonimizados de múltiplos países. Essa rede é composta por organizações em saúde ( *Healthcare Organizations - HCOs* ), incluindo centros acadêmicos, centros médicos especializados e hospitais comunitários. Os dados estão concentrados no DataLab da Inovação, Pesquisa e Ensino do Hospital Alemão Oswaldo Cruz (HAOC), em São Paulo, representando instituição *hub* da TriNetX no Brasil.

Foram avaliados mulheres e homens com diagnóstico confirmado de IAM, admitidos em hospitais componentes da rede colaborativa no Brasil (23 instituições), inseridos na plataforma TriNetX pela primeira ocorrência do evento agudo, retrospectivamente dentro de 10 anos (2013-2023), com seguimento mínimo de 5 anos após a alta hospitalar do primeiro IAM. Analisamos todos os pacientes do sexo feminino ou masculino, com diagnóstico por CID-11 código I21 (IAM), e idade ≥18 anos, com tempo mínimo de 5 anos de dados de seguimento disponíveis na coorte brasileira da plataforma TriNetX. Analisamos as seguintes variáveis: Fatores de risco cardiovascular e comorbidades (diagnósticos por CID-11); manifestações clínicas; exames laboratoriais; propedêutica realizada e terapia farmacológica prescrita.

### Desfechos analisados

Os desfechos clínicos foram avaliados após a fase hospitalar e até 5 anos (óbito, nova hospitalização por IAM, procedimentos de revascularização miocárdica, ou insuficiência cardíaca).

### Cálculo do tamanho amostral e análise estatística

**Figura 1:**
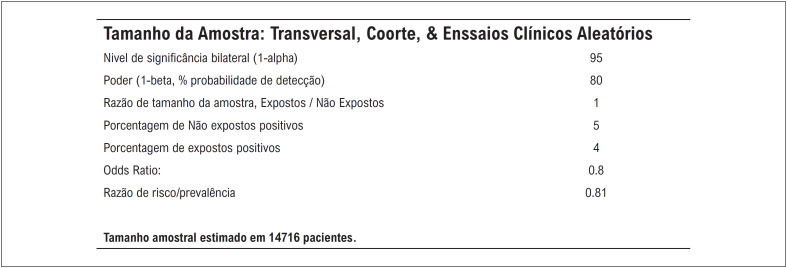
Cálculo do Tamanho da Amostra

O cálculo do tamanho amostral foi desenvolvido em conjunto com o Laboratório de Estatística e Epidemiologia local. Utilizamos os dados de proporção de eventos esperados e *Odds Ratio* para cálculo estimado inicial. De fato, com o tamanho amostral já englobando praticamente 100% dos pacientes, com mínima proporção de dados incompletos sobre ocorrência dos eventos, estimamos que o tamanho amostral a ser incluído tivesse as premissas estatísticas apresentadas na Figura 1 .

O nível de significância estatística adotado na análise foi de 5% (0,05). Utilizamos testes de significância bilaterais. As variáveis contínuas foram expressas através de média ± desvio-padrão, pois apresentaram distribuição normal. As análises de comparações destas variáveis entre os grupos mulheres versus homens foram efetuadas por teste t-Student não pareado. Para testar a normalidade, utilizamos o teste de Shapiro-Wilk. As distribuições das variáveis categóricas foram expressas como frequências e porcentagens, e as respectivas comparações foram realizadas por teste do χ^2^ ou teste exato de Fisher, conforme apropriado. A análise dos desfechos foi conduzida com base no tempo para ocorrência do evento primário, e curvas de sobrevida livre de eventos e mortalidade total por método de Kaplan-Meier, com teste de log-rank para significância estatística entre as curvas de distribuição. Os programas estatísticos utilizados foram o sistema R e o SPSS Statistics versão 19.0, além de 4 programas ou ferramentas de linguagem intrínsecas ao sistema da plataforma TriNetX.

## Resultados

Por meio de busca sistematizada na plataforma TriNetX, foram identificados 29.041 pacientes, dos quais 17.757 (61,2%) homens e 11.284 (38,8%) mulheres, hospitalizados com o diagnóstico de primeiro IAM. A idade média da população feminina foi 64,4 ± 14,5 anos e da população masculina, 59,8 ± 13,4 anos (p < 0,001). As comorbidades da população estudada encontram-se descritas na Tabela 1 .

**Tabela 1:** Comorbidades

Comorbidade	Mulheres – n (%)	Homens – n (%)	Valor de p
Hipertensão	1.552 (13,8%)	1.639 (9,1%)	< 0,001
Diabetes Mellitus	523 (4,6%)	681 (3,8%)	< 0,001
Sobrepeso e Obesidade	73 (0,6%)	73 (0,4%)	0,005
Dislipidemia	50 (0,4%)	86 (0,5%)	0,661
Doença Renal Crônica	214 (1,9%)	322 (1,8%)	0,521
Neoplasias	464 (4,1%)	548 (3,1%)	< 0,001

As mulheres apresentaram com maior frequência os sintomas conjuntos de dor cervical e torácica, além de sintomas respiratórios como tosse e infecções agudas de vias aéreas superiores em comparação aos homens ( Tabela 2 ).

**Tabela 2:** Manifestações clínicas

Sintoma	Mulheres – n (%)	Homens – n (%)	Valor de p
Dor cervical e torácica	2.194 (19,4%)	2.816 (15,7%)	< 0,001
Tosse	659 (5,8 %)	623 (3,5%)	< 0,001
Infecções Agudas de Vias Aéreas Superiores	1.711 (15,2 %)	2.167 (12,1%)	< 0,001

As mulheres apresentaram níveis sérios significativamente mais baixos de creatinina e de troponina do que os valores observados em homens. Por outro lado, apresentaram valores de colesterol total e LDL mais elevados. Os homens apresentaram maiores níveis séricos de e de hemoglobina.

Não houve diferença significativa em relação ao perfil glicêmico, com mulheres e homens apresentando níveis séricos semelhantes de glicemia e de hemoglobina glicada ( Tabela 3 ).

**Tabela 3:** Parâmetros laboratoriais

Parâmetro	Mulheres Média ± (DP)	Homens Média ± DP)	Valor de p
Creatinina (mg/dL)	1,0 ± 0,9	1,2 ± 1,1	< 0,001
Troponina I (ng/mL)	3,8 ± 18,2	5,5 ± 18	< 0,001
Colesterol Total (mg/dL)	192,3 ± 53,1	181 ± 54,6	< 0,001
Colesterol LDL (mg/dL)	115,9 ± 45,6	109,4 ± 44,7	< 0,001
Colesterol HDL (mg/dL)	48,3 ± 13,4	40 ± 10,7	< 0,001
Triglicérides (mg/dL)	160,0 ± 102,8	184,1 ± 200,1	< 0,001
Glicemia (mg/dL)	128,7 ± 65,2	131 ± 66,5	0,057
Hemoglobina glicada (%)	6,9 ± 1,9	6,9 ± 1,9	0,304
Hemoglobina (g/dL)	12,3 ± 1,9	13,5 ± 2,3	< 0,001
Leucócitos (céls./mm³)	3.992 ± 1.250,7	3.140 ± 1.120	< 0,001
Plaquetas (10³/mm³)	264 ± 87,4	237,1 ± 77,6	< 0,001

* representados por média e desvio padrão.

Observamos menor realização de teste ergométrico, cintilografia de perfusão miocárdica e ventriculografia esquerda invasiva em pacientes do sexo feminino. Não houve diferença significativa na realização de ecocardiograma transtorácico em repouso, ecocardiograma de estresse, ou ressonância magnética do coração ( Tabela 4 ).

**Tabela 4:** Propedêutica e procedimentos realizados

Procedimento	Mulheres – n (%)	Homens – n (%)	Valor de p
Ecocardiograma	1.648 (14,6%)	2.542 (14,2%)	0,286
Teste Ergométrico	169 (1,5%)	523 (2,9 %)	< 0,001
Ecocardiograma com Estresse	10 (0,1%)	10 (0,1%)	0,294
Cintilografia Miocárdica	147 (1,3%)	337 (1,9%)	< 0,001
Ressonância Magnética de Coração	47 (0,4%)	66 (0,4%)	0,511
Ventriculografia	806 (7,1%)	1.146 (8,1%)	0,005

Identificamos, no grupo de mulheres, menor prescrição de antiplaquetários (aqui descritos na forma de uso isolado), i.e., ácido acetilsalicílico, clopidogrel e prasugrel. Por outro lado, foram prescritos mais frequentemente inibidores da enzima conversora de angiotensina, bloqueadores do receptor de angiotensina II, e antagonistas de canal de cálcio. Não houve diferença significativa entre os grupos em relação à prescrição de hipolipemiantes, betabloqueadores e antianginosos ( Tabela 5 ).

**Tabela 5:** Terapia farmacológica administrada na internação

Medicamento ou Classe Medicamentosa	Mulheres – n (%)	Homens – n (%)	Valor de p
AAS	4.573 (40,5%)	7.376 (41,5%)	0,045
Clopidogrel	3.263 (28,9 %)	5.534 (30,8%)	0,001
Ticagrelor	210 (1,9%)	379 (2,1%)	0,139
Prasugrel	18 (0,2%)	73 (0,4%)	< 0,001
Hipolipemiantes	1.656 (14,7%)	2.591 (14,4%)	0,560
Inibidores da Enzima Conversora de Angiotensina	3.294 (29,2%)	4.567 (25,4%)	< 0,001
Bloqueadores do Receptor de Angiotensina II	1.300 (11,5%)	1.651 (9,2%)	< 0,001
Betabloqueadores	2.676 (23,7%)	4.417 (24,6%)	0,086
Diuréticos	2.853 (25,3%)	3.750 (20,9%)	< 0,001
Antianginosos	1.707 (15,1%)	2.782 (15,5%)	0,399
Bloqueadores de Canal de Cálcio	1.260 (11,2%)	1.392 (7,8 %)	< 0,001

**Figura 2:**
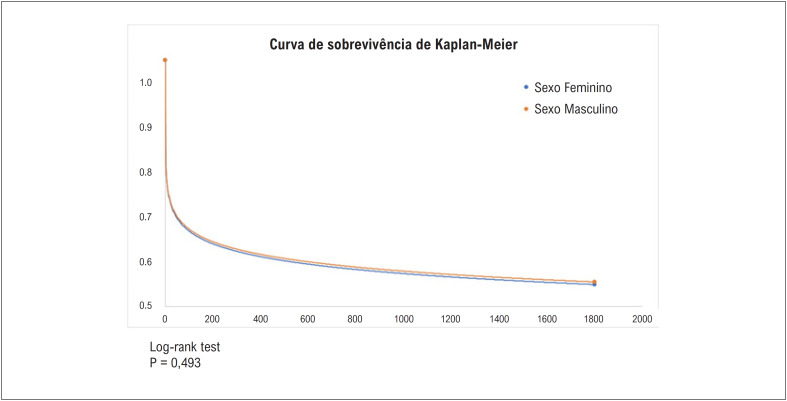
Curva de Sobrevivência de Kaplan-Meier (em dias de observação).

O grupo de mulheres apresentou maior ocorrência do desfecho combinado de óbito, nova hospitalização por IAM, procedimentos de revascularização miocárdica, ou insuficiência cardíaca após fase hospitalar e com seguimento de 5 anos ( Tabela 6 e Figura 2 ).

**Tabela 6:** Desfecho composto por óbito, nova hospitalização por IAM, procedimentos de revascularização miocárdica, ou insuficiência cardíaca após fase hospitalar e com seguimento de 5 anos

Sexo	Nº de Pacientes	Nº de Pacientes com Evento	Odds Ratio	IC 95%	Valor de p
Feminino	11.284	3.592	1,058	(1,005 – 1,113)	0,03
Masculino	17.957	5.500			

## Discussão

Ao nosso conhecimento e pela evidência disponível, este estudo representa a maior coorte brasileira já avaliada e com maior tempo de seguimento após a ocorrência do primeiro IAM, com foco específico na comparação entre mulheres e homens. Avaliamos a base de dados referente aos pacientes hospitalizados e com dados inseridos nos prontuários eletrônicos das 23 instituições que compõem a base do país na plataforma colaborativa multicêntrica e internacional TriNetX. Comparamos 17.757 homens e 11.284 mulheres hospitalizados com diagnóstico do primeiro IAM quanto à ocorrência dos desfechos cardiovasculares após o evento índice no longo prazo, com acompanhamento mínimo de 5 anos após alta hospitalar. Os resultados da coorte analisada ressaltam que as mulheres apresentaram perfil clínico com aspectos de maior morbidade e maior risco subsequente à alta hospitalar no primeiro IAM para eventos cardiovasculares combinados no longo prazo ( Figura Central ).

Os dados deste estudo compreendem informações do mundo real, obtidas a partir de pacientes hospitalizados em diversos centros de saúde, que incluem instituições acadêmicas, privadas ou filantrópicas. Essa abordagem abrange uma população diversa do ponto de vista sociodemográfico e de complexidade clínica heterogênea, proporcionando uma representação fidedigna do contexto de saúde brasileiro.

Em consonância com estudos prévios, observamos que a população feminina desta coorte apresenta prevalência significativamente maior de comorbidades que conferem maior risco cardiovascular, como hipertensão, diabetes, sobrepeso e obesidade, o que pode justificar, parcialmente, a diferença de prognóstico encontrada entre mulheres e homens. Não foram observadas, entretanto, diferenças significativas entre os grupos em relação ao diagnóstico prévio de doença renal crônica e de dislipidemia, achado que difere de outras coortes internacionais, o que pode ser explicado por diferenças em características sociodemográficas, de hábitos dietéticos e de atividade física, e de perfil antropométrico. Apesar disso, em exames laboratoriais realizados durante a fase hospitalar, identificamos níveis séricos mais elevados de LDL colesterol em mulheres, o que sugere a possibilidade de subdiagnóstico de dislipidemia nessa população ou de menores índices de tratamento farmacológico adequado com hipolipemiantes, particularmente estatinas.

No contexto da apresentação clínica na avaliação inicial do primeiro IAM, observamos uma maior prevalência de sintomas respiratórios entre as mulheres. Esse achado é, também, consistente com a literatura internacional, que evidencia diferenças significativas nas manifestações clínicas de apresentação de IAM entre os sexos.^17 , 18^ Outro ponto de concordância deste estudo em relação à evidência prévia foi a observação de menores níveis séricos de troponina I no sexo feminino. A hipótese é de que haja maior proporção de IAM sem elevação do segmento ST, com trombos coronários não oclusivos, maior proporção de erosões ao invés de roturas de placas ateroscleróticas, ou DAC multiarterial e presença de maior número de colaterais, o que pode, em conjunto e de forma multifatorial, promover menor impacto em termos de magnitude de necrose miocárdica, portanto, menores elevações dos níveis de troponina cardíaca.

Apesar do benefício bem estabelecido de medicações comprovadas no tratamento do IAM, houve menor prescrição documentada de dupla antiagregação plaquetária, o que reforça achados de outros relatos, e pode estar relacionado à maior morbimortalidade observada nesse grupo. De fato, conforme demonstrado em estudos prévios, as pacientes que não receberam terapia antiplaquetária adequada no momento da alta evoluem com maior risco de readmissão hospitalar e óbito. Uma explicação plausível para esse achado reside na maior prevalência de DAC não obstrutiva (<50% de estenose luminal) entre as mulheres. Nesse contexto, o papel da terapia de dupla antiagregação plaquetária e sem a realização de intervenção coronária percutânea com implante de stents se associa a menor magnitude de benefício, especificamente pela ausência de redução do desfecho de mortalidade (isolado) com uso de clopidogrel (inibidor P2y12 mais prescrito globalmente e no Brasil) e ácido acetilsalicílico.

Por outro lado, em contraste com relatos em outras populações, observou-se índices semelhantes na prescrição de medicações hipolipemiantes entre os sexos, indicando uma abordagem mais equilibrada nesta corte. Além disso, destacamos a prescrição de inibidores do sistema renina-angiotensina-aldosterona de modo mais frequente na população feminina, possivelmente devido à maior incidência de hipertensão e diabetes mellitus nesse grupo.

A análise abrangente desta extensa coorte de pacientes brasileiros revela consistência com a literatura internacional. Identificamos não apenas o aumento do risco de desfechos cardiovasculares desfavoráveis em mulheres após o evento índice no acompanhamento de longo prazo, mas também à identificação de discrepâncias entre as populações em diversos aspectos, como os fatores de risco cardiovasculares, manifestações clínicas à admissão e a administração de terapias preconizadas por recomendações classe I nível A em diretrizes ao longo do tempo de observação desta coorte.

A doença coronariana, especificamente a síndrome instável aguda aqui estudada —IAM—, foi historicamente associada ao sexo masculino. Entretanto, conforme amplamente documentado na literatura e respaldado nesta coorte, é evidente que essa condição também representa uma das principais causas de morbidade e mortalidade entre as mulheres, com gravidade maior nesse grupo.^19 , 20^ Este achado ressalta a relevância e a urgência de promover a educação multiprofissional continuada e de implementar medidas direcionadas para atenuar essa disparidade observada e aprimorar o prognóstico do IAM na população feminina.

## Limitações

A principal limitação deste estudo é sua natureza observacional, uma vez que a comparação entre os sexos está sujeita à presença de fatores de confusão ou não mensurados pela própria ausência de documentação em prontuários, o que pode influenciar a ocorrência dos desfechos clínicos de interesse. Outra limitação do estudo diz respeito à sua natureza retrospectiva, que depende da coleta de dados a partir de prontuários eletrônicos. Nesse contexto, a precisão das informações está sujeita à correta inserção de dados estruturados por parte dos profissionais de saúde, o que pode resultar em dados incompletos ou ausentes, fato observado em pesquisas de mundo real baseadas em dados inseridos espontaneamente e não em formulários de coleta de dados especificamente elaborados para tal.

## Conclusões

Em uma grande coorte brasileira derivada do mundo real, com o propósito de comparar mulheres e homens hospitalizados após o primeiro IAM, observamos a incidência mais elevada dos desfechos cardiovasculares clinicamente relevantes entre as mulheres, incluindo mortalidade, novas hospitalizações por IAM, procedimentos de revascularização miocárdica e insuficiência cardíaca no longo prazo após a alta hospitalar do evento índice.

Identificamos maior prevalência de comorbidades relacionadas ao alto risco cardiovascular, como hipertensão, diabetes e obesidade entre as mulheres, em comparação aos homens.

Outros achados relevantes incluíram uma frequência mais alta de apresentação atípica dos sintomas iniciais do evento clínico agudo e uma menor prescrição de terapia farmacológica baseada em evidências, com destaque para a subutilização da dupla antiagregação plaquetária, mesmo após pareamento por escore de propensão.
