# Supplementary material for: Fatores de Risco, Manejo e Evolução após Primeiro Infarto Agudo do Miocárdio: Um Estudo de Mundo Real Comparando Coortes de Mulheres e Homens na Rede TriNetX
Source: Arq Bras Cardiol. 2024 Oct 17;121(10):e20230692. [Article in Portuguese] doi: 10.36660/abc.20230692 (PMC11634215; doi:10.36660/abc.20230692)
Supplement: Translation

# Translation: Risk Factors, Management, and Evolution after the First Acute Myocardial Infarction: A Real-World Study Comparing Cohorts of Women and Men in the TriNetX Network

**DOI:** 10.36660/abc.20230692i

**Authors:** Camila Mota Guida, Eduardo Juvenal de Souza, Leandro Menezes Alves da Costa, Thiago Luis Scudeler, Rafael Amorim Belo Nunes, Gustavo Bernardes de Figueiredo Oliveira

**Affiliations:** 1 São Paulo SP Brazil Instituto Dante Pazzanese de Cardiologia São Paulo, SP – Brazil; 2 São Paulo SP Brazil Hospital Alemão Oswaldo Cruz, São Paulo, SP – Brazil

**Keywords:** Acute Myocardial Infarction, Risk Factors, Cardiovascular Disease in Women

## Abstract

**Background:**

International cohort studies have consistently demonstrated an unfavorable prognosis in female patients after the first acute myocardial infarction (AMI) over the past decades. However, national data on this topic are limited.

**Objectives:**

This study aims to compare national cohorts of men and women hospitalized due to the first acute myocardial infarction, examining long-term outcomes.

**Methods:**

A retrospective, observational study using real-world data extracted from the global TriNetX platform, including patients of both sexes with a confirmed diagnosis of AMI according to the International Classification of Diseases (ICD), version 11, code I21. The level of statistical significance adopted in the analysis was 5% (0.05). The primary outcome assessed was a composite of death, new hospitalization for AMI, myocardial revascularization procedures, or heart failure after the hospital phase with a 5-year follow-up.

**Results:**

Data from 29,041 patients were evaluated, of which 11,284 (38.4%) were women. The mean age of the female and male populations was 64.4 and 59.8 years, respectively. The group of women showed a higher occurrence of the composite outcome of death, new hospitalization for AMI, myocardial revascularization procedures, or heart failure after the hospital phase with a 5-year follow-up (OR 1.058; CI 1.005 - 1.113; p = 0.03).

**Conclusions:**

In this large Brazilian cohort, the female sex was associated with a higher occurrence of cardiovascular events within 5 years after hospital discharge.

## Introduction

Currently, coronary atherosclerotic disease is the leading cause of death and loss of disability-adjusted life years (DALYs) worldwide, accounting for nearly 7 million deaths and 129 million DALYs per year.^1^In Brazil specifically, about 13% of deaths can be attributed to coronary artery disease (CAD), according to 2017 data.^2^

Despite significant improvements in cardiovascular mortality among women over the past two decades, coronary disease remains understudied, underdiagnosed, and undertreated in this group.^3 , 4^The disparity between men and women is evident in all contexts: pre-hospital care; time elapsed before seeking emergency care; clinical manifestations of the acute event; identified risk factors; characteristics of coronary lesions; time to initiation of specific treatment; indication for revascularization; post-discharge care, and prognosis in both the short and long term.^5 - 10^The low representation of women in the scientific evidence that guides cardiological practices also stands out as a possible contributor to the observed discrepancies.^11 - 16^

Therefore, it is essential to generate knowledge about the risk factors, signs and symptoms, angiographic characteristics, hospital management, and prognostic factors between women and men, especially in the context of the first acute myocardial infarction (AMI), a scenario of significant morbidity and mortality that often affects patients who are vulnerable due to lack of awareness of their own cardiovascular risk.

## Objectives

Based on the gaps in national and contemporary evidence in robust comparisons between women and men with a first AMI, and considering the relevance of health equity in the Brazilian population, we established the following objectives: 1. To compare women and men diagnosed with a first AMI regarding the risk of composite cardiovascular outcomes after the hospital phase, up to 5 years (death, new hospitalization for AMI, new myocardial revascularization procedures, or heart failure); 2. To compare the contemporary clinical profile in terms of comorbidities and clinical presentation in the context of the first AMI.

## Methods

### Study design

This is an observational study with a retrospective cohort and cross-sectional analysis of women and men with a confirmed diagnosis of AMI. We used data from the global collaborative real-world evidence platform TriNetX, a global health research network that is updated weekly with data recorded in anonymized electronic medical records from multiple countries. This network comprises Healthcare Organizations (HCOs), including academic centers, specialized medical centers, and community hospitals. The data are centralized in the DataLab for Innovation, Research, and Education at Hospital Alemão Oswaldo Cruz (HAOC) in São Paulo, which serves as the hub institution for TriNetX in Brazil.

Women and men with a confirmed diagnosis of AMI admitted to hospitals within the collaborative network in Brazil (23 institutions) were evaluated. These patients were included in the TriNetX platform for the first occurrence of the acute event, retrospectively within 10 years (2013-2023), with a minimum follow-up of 5 years after discharge from the first AMI. We analyzed all female and male patients with an ICD-11 code I21 (AMI) diagnosis, aged ≥18 years, with a minimum of 5 years of follow-up data available in the Brazilian cohort on the TriNetX platform. The following variables were analyzed: Cardiovascular risk factors and comorbidities (ICD-11 diagnoses), clinical manifestations, laboratory tests, procedures performed, and prescribed pharmacological therapy.

### Outcomes analyzed

The clinical outcomes were evaluated after the hospital phase and up to 5 years (death, new hospitalization for AMI, myocardial revascularization procedures, or heart failure).

### Sample size calculation and statistical analysis

The sample size calculation was developed in collaboration with the local Statistics and Epidemiology Laboratory. We used the data on the expected proportion of events and the Odds Ratio (OR) for the initial estimated calculation. Indeed, with the sample size already encompassing nearly 100% of the patients, with minimal proportion of incomplete data on the occurrence of events, we estimated that the sample size to be included met the statistical assumptions presented in Figure 1 .

**Figure 1:** Sample Size Calculation.

The level of statistical significance adopted in the analysis was 5% (0.05). We used two-tailed significance tests. Continuous variables were expressed as mean ± standard deviation, as they presented a normal distribution. Comparisons of these variables between women and men were performed using an unpaired t-test. The Shapiro-Wilk test was used to test normality. Categorical variables were expressed as frequencies and percentages, and the respective comparisons were performed using the χ2 test or Fisher's exact test, as appropriate. Outcome analysis was conducted based on the time to the occurrence of the primary event, and event-free survival and total mortality curves were generated using the Kaplan-Meier method, with a log-rank test for statistical significance between the distribution curves. The statistical programs used were the R system and SPSS Statistics version 19.0, in addition to four intrinsic language programs or tools within the TriNetX platform.

## Results

Through a systematic search on the TriNetX platform, 29,041 patients were identified, of whom 17,757 (61.2%) were men and 11,284 (38.8%) were women hospitalized with a diagnosis of a first AMI. The mean age of the female population was 64.4 ± 14.5 years, and the mean age of the male population was 59.8 ± 13.4 years (p < 0.001). The comorbidities of the studied population are described in Table 1 .

**Table 1:** Comorbidities

Comorbidity	Women – n (%)	Men – n (%)	p-value
Hypertension	1,552 (13.8%)	1,639 (9.1%)	< 0.001
Diabetes Mellitus	523 (4.6%)	681 (3.8%)	< 0.001
Overweight and Obesity	73 (0.6%)	73 (0.4%)	0.005
Dyslipidemia	50 (0.4%)	86 (0.5%)	0.661
Chronic Kidney Disease	214 (1.9%)	322 (1.8%)	0.521
Cancer	464 (4.1%)	548 (3.1%)	< 0.001

Women more frequently presented with a combination of neck and chest pain, as well as respiratory symptoms such as cough and acute upper respiratory tract infections, compared to men ( Table 2 ).

**Table 2:** Clinical Manifestations

Symptom	Women – n (%)	Men – n (%)	p-value
Neck and chest pain	2,194 (19.4%)	2,816 (15.7%)	< 0.001
Cough	659 (5.8 %)	623 (3.5%)	< 0.001
Acute Upper Respiratory Tract Infections	1,711 (15.2 %)	2,167 (12.1%)	< 0.001

Women presented significantly lower serum levels of creatinine and troponin than the values observed in men. On the other hand, they had higher levels of total and LDL cholesterol. Men showed higher serum levels of hemoglobin. There was no significant difference regarding the glycemic profile, with women and men presenting similar serum levels of glucose and glycated hemoglobin ( Table 3 ).

**Table 3:** Laboratory Parameters

Parameter	Women - Mean ± SD	Men - Mean ± SD	p-value
Creatinine (mg/dL)	1.0 ± 0.9	1.2 ± 1.1	< 0.001
Troponin I (ng/mL)	3.8 ± 18.2	5.5 ± 18	< 0.001
Total Cholesterol (mg/dL)	192.3 ± 53.1	181 ± 54.6	< 0.001
LDL Cholesterol (mg/dL)	115.9 ± 45.6	109.4 ± 44.7	< 0.001
HDL Cholesterol (mg/dL)	48.3 ± 13.4	40 ± 10,7	< 0.001
Triglycerides (mg/dL)	160.0 ± 102.8	184.1 ± 200.1	< 0.001
Glucose (mg/dL)	128.7 ± 65.2	131 ± 66.5	0.057
Glycated Hemoglobin (%)	6.9 ± 1.9	6.9 ± 1.9	0.304
Hemoglobin (g/dL)	12.3 ± 1.9	13.5 ± 2.3	< 0.001
Leukocytes (cells/mm³)	3,992 ± 1,250.7	3,140 ± 1,120	< 0.001
Platelets (10³/mm³)	264 ± 87.4	237.1 ± 77.6	< 0.001

We observed a lower frequency of stress tests, myocardial perfusion scintigraphy, and invasive left ventriculography in female patients. There was no significant difference in the performance of resting transthoracic echocardiograms, stress echocardiograms, or cardiac magnetic resonance imaging ( Table 4 ).

**Table 4:** Diagnostic and Procedural Assessments

Procedure	Women – n (%)	Men – n (%)	p-value
Transthoracic Echocardiogram	1,648 (14.6%)	2,542 (14.2%)	0.286
Stress Test	169 (1.5%)	523 (2.9 %)	< 0.001
Stress Echocardiogram	10 (0.1%)	10 (0.1%)	0.294
Myocardial Scintigraphy	147 (1.3%)	337 (1.9%)	< 0.001
Cardiac Magnetic Resonance Imaging	47 (0.4%)	66 (0.4%)	0.511
Ventriculography	806 (7.1%)	1,146 (8.1%)	0.005

We identified lower prescription rates of antiplatelet drugs (listed here as single-use), such as aspirin, clopidogrel, and prasugrel, among the female group. Conversely, angiotensin-converting enzyme (ACE) inhibitors, angiotensin II receptor blockers, and calcium channel antagonists were prescribed more frequently. There were no significant differences between the groups regarding the prescription of lipid-lowering agents, beta-blockers, and antianginals ( Table 5 ).

**Table 5:** Pharmacological Therapy Administered During Hospitalization

Medication or Drug Class	Women – n (%)	Men – n (%)	p-value
Aspirin (AAS)	4,573 (40.5%)	7,376 (41.5%)	0.045
Clopidogrel	3,263 (28.9 %)	5,534 (30.8%)	0.001
Ticagrelor	210 (1.9%)	379 (2.1%)	0.139
Prasugrel	18 (0.2%)	73 (0.4%)	< 0.001
Lipid-lowering Agents	1,656 (14.7%)	2,591 (14.4%)	0.560
ACE Inhibitors	3,294 (29.2%)	4,567 (25.4%)	< 0.001
Angiotensin II Receptor Blockers	1,300 (11.5%)	1,651 (9.2%)	< 0.001
Beta-blockers	2,676 (23.7%)	4,417 (24.6%)	0.086
Diuretics	2,853 (25.3%)	3,750 (20.9%)	< 0.001
Antianginals	1,707 (15.1%)	2,782 (15.5%)	0.399
Calcium Channel Blockers	1,260 (11.2%)	1,392 (7.8 %)	< 0.001

The female group exhibited a higher incidence of the composite outcome of death, new hospitalization for AMI, myocardial revascularization procedures, or heart failure after hospital discharge and with 5 years of follow-up ( Table 6 and Figure 2 ).

**Table 6:** Composite Outcome of Death, New Hospitalization for AMI, Myocardial Revascularization Procedures, or Heart Failure After Hospital Discharge and 5-Year Follow-Up

Sex	Number of Patients	Number of Patients with Event	Odds Ratio	95% CI (Confidence Interval)	p-value
Female	11,284	3,592	1.058	(1.005 – 1.113)	0.03
Male	17,957	5,500			

**Figure 2:** Kaplan-Meier Survival Curve (in days of observation).

## Discussion

To our knowledge and based on available evidence, this study represents the largest Brazilian cohort ever evaluated with the longest follow-up after the first AMI, specifically focusing on the comparison between women and men. We analyzed data from hospitalized patients with clinical data entered into the electronic health records of the 23 institutions that comprise the national database on the multicentric and international collaborative platform TriNetX. We compared 17,757 men and 11,284 women hospitalized with a diagnosis of first AMI regarding the occurrence of cardiovascular outcomes after the index event in the long term, with a minimum follow-up of 5 years after hospital discharge. The results of the analyzed cohort emphasize that women had a clinical profile with aspects of greater morbidity and higher risk subsequent to hospital discharge after the first AMI for combined cardiovascular events in the long term (Central Illustration).

The data from this study include real-world information obtained from hospitalized patients across various healthcare centers, including academic, private, and philanthropic institutions. This approach encompasses a diverse population in terms of sociodemographic factors and clinical complexity, providing an accurate representation of the Brazilian health context.

In line with previous studies, we observed that the female population in this cohort has a significantly higher prevalence of comorbidities that confer greater cardiovascular risk, such as hypertension, diabetes, overweight, and obesity. This may partially explain the prognostic differences found between women and men. However, no significant differences were observed between the groups regarding the prior diagnosis of chronic kidney disease and dyslipidemia. This finding differs from other international cohorts and may be explained by differences in sociodemographic characteristics, dietary habits, physical activity, and anthropometric profiles. Despite this, laboratory tests performed during the hospital phase identified higher serum levels of LDL cholesterol in women, suggesting the possibility of underdiagnosis of dyslipidemia in this population or lower rates of appropriate pharmacological treatment with lipid-lowering agents, particularly statins.

Regarding clinical presentation at the initial evaluation of the first AMI, we observed a higher prevalence of respiratory symptoms among women. This finding is also consistent with international literature, which highlights significant differences in the clinical manifestations of AMI presentation between sexes.^17 , 18^Another agreement with previous evidence was the observation of lower serum levels of troponin I in women. The hypothesis is that there may be a higher proportion of non-ST-elevation AMIs, non-occlusive coronary thrombi, a higher proportion of erosions rather than ruptures of atherosclerotic plaques, or multi-arterial coronary artery disease with a higher number of collaterals, which may, collectively and multifactorially, contribute to a lesser impact in terms of myocardial necrosis magnitude, and therefore, lower elevations in cardiac troponin levels.

Despite the well-established benefits of proven medications in the treatment of AMI, there was less documented prescription of dual antiplatelet therapy, reinforcing findings from other reports, and this may be related to the higher morbidity and mortality observed in this group. Indeed, as demonstrated in previous studies, patients who did not receive adequate antiplatelet therapy at discharge have a higher risk of readmission and death. A plausible explanation for this finding lies in the higher prevalence of non-obstructive coronary artery disease (<50% lumen stenosis) among women. In this context, the role of dual antiplatelet therapy, without percutaneous coronary intervention with stent implantation, is associated with a lesser degree of benefit, specifically due to the lack of reduction in mortality outcomes (isolated) with the use of clopidogrel (the most globally and nationally prescribed P2Y12 inhibitor) and aspirin.

On the other hand, in contrast to reports from different populations, similar rates of lipid-lowering medication prescriptions were observed between sexes, indicating a more balanced approach in this cohort. Furthermore, we highlight the more frequent prescription of renin-angiotensin-aldosterone system inhibitors in the female population, possibly due to the higher incidence of hypertension and diabetes mellitus in this group.

The comprehensive analysis of this extensive Brazilian cohort reveals consistency with international literature. We identified not only an increased risk of adverse cardiovascular outcomes in women after the index event in long-term follow-up but also discrepancies between populations in various aspects, such as cardiovascular risk factors, clinical manifestations at admission, and administration of therapies recommended by Class I Level A guidelines over the observation period of this cohort.

Coronary artery disease, specifically acute unstable syndrome studied here—AMI—has historically been associated with the male sex. However, as widely documented in the literature and supported by this cohort, it is evident that this condition also represents one of the main causes of morbidity and mortality among women, with greater severity in this group.^19 , 20^This finding underscores the relevance and urgency of promoting continued multidisciplinary education and implementing targeted measures to address this observed disparity and improve the prognosis of AMI in the female population.

## Limitations

The main limitation of this study is its observational nature, as comparisons between sexes are subject to the presence of confounding factors or unmeasured factors due to the lack of documentation in medical records, which may influence the occurrence of clinical outcomes of interest. Another limitation is its retrospective nature, which depends on data collection from electronic health records. In this context, the accuracy of information is subject to the correct entry of structured data by healthcare professionals, which may result in incomplete or missing data, a fact observed in real-world research based on spontaneously entered data rather than data collection forms specifically designed for this purpose.

## Conclusions

In a large real-world Brazilian cohort aimed at comparing women and men hospitalized after their first AMI, we observed a higher incidence of clinically relevant cardiovascular outcomes among women, including mortality, new hospitalizations for AMI, myocardial revascularization procedures, and heart failure in the long term after hospital discharge from the index event.

We identified a higher prevalence of comorbidities related to high cardiovascular risk, such as hypertension, diabetes, and obesity, among women compared to men.

Other relevant findings included a higher frequency of atypical presentation of initial symptoms of the acute clinical event and a lower prescription of evidence-based pharmacological therapy, with particular emphasis on the underutilization of dual antiplatelet therapy, even after propensity score matching.
